# William James and British thought: then and now

**DOI:** 10.1192/bjb.2019.56

**Published:** 2020-04

**Authors:** David E. Leary

**Affiliations:** School of Arts and Sciences, University of Richmond, USA

**Keywords:** William James, history of psychology, panic disorders, attention and will

## Abstract

The American psychologist and philosopher William James drew inspiration from British evolutionary theory, neurology, psychiatry, psychology and philosophy. Trained in anatomy, physiology and medicine, he developed a physiological psychology that offered acute analyses of consciousness and of the relations between mind and brain, habit and thought, cognition and emotion and other aspects of psychology. One of his insights, regarding the relation between attention and will, was based upon his own experience of panic anxiety, which was resolved through his reading of several British authors. The story of his psychiatric experience, practical response and later theoretical conclusion offers a potential contribution to contemporary therapeutic practice.

William James (1842–1910) was and remains one of the leading academic and public intellectuals in American history. A founder of modern scientific psychology, he was also instrumental in orienting contemporary clinical psychology. While establishing the pragmatic tradition in American philosophy, he became an inspiring and popular public lecturer, addressing topics like the will to believe and what makes life worth living. Although a psychologist and philosopher, he had a much broader background that included other fields in the sciences and humanities, but for all of that, the only degree he ever received was an MD from Harvard Medical School. His first job at Harvard, before transferring to its Department of Philosophy, was as an Instructor of Anatomy and Physiology.

James's significant contributions to the history of psychology and philosophy depended upon his knowledge of German philosophy and experimental psychology as well as his familiarity with French philosophy and clinical psychiatry, but just as important was his intimate connection with British philosophy and psychology, and his earlier study of British physiology, medical psychology and psychiatry. Beyond that, Charles Darwin provided the larger conceptual framework for his work, British neurologists helped him understand the workings of the brain and many other British thinkers, including literary authors, contributed insights that he integrated into his monumental *Principles of Psychology* (1890)^1^ as well as into his later philosophical work. Indeed, he explicitly stated that his psychology represented a continuation, albeit with important revisions, of the tradition that began with John Locke's empirical psychology, and he dedicated his ground-breaking philosophical work, *Pragmatism* (1907),^2^ to ‘the Memory of John Stuart Mill’, from whom he had learned ‘pragmatic openness of mind’.

It was from Britain, too, that James received an invitation to deliver lectures, in Edinburgh, that led to his *Varieties of Religious Experience* (1902),^3^ which is still the starting point for empirical studies of the subjective aspects of religious belief, feeling and action. And just a few years later, Oxford invited him to deliver the lectures on *A Pluralistic Universe* (1909)^4^ in which he called for respectful awareness and acceptance of the diverse array of people, phenomena, perspectives and persuasions that compose this world of ours. (He had earlier denounced ‘blindness’ to the valuable distinctiveness of those unlike ourselves, citing Robert Louis Stevenson as well as Wordsworth to bolster his argument.)

Bringing together German, French and British sources was not the only act of integration that made James's *Principles of Psychology* such a landmark achievement. He also joined the scientific with the humanistic dimensions of psychology. His central argument was that the human mind depends upon, but is not reducible to, its physiological and neurological conditions. To the contrary, it is an essential factor in the evolution and operation of those conditions, a proposition that is once again being entertained by many, after decades of neglect. Ironically, James's conjoining of physiology and neurology with a careful and precise phenomenology of consciousness drew innumerable readers to his work, but also set the scene for the future dismemberment of what he had brought together. For he was so successful in drawing attention to the significance of the nervous system for an understanding of psychological phenomena that he helped to generate a thriving tradition of neuropsychological research that, until recently, overlooked the reciprocal influence of consciousness on the human organism, which was equally important to James. Similarly, James's acute analysis of habit helped to inspire generations of behaviourists who proceeded, until the past quarter-century, to ignore the essential role of thought in the development and modification of habits, which James had strongly emphasised. And, on the other side of the coin, those who loved his masterful descriptions of consciousness, thinking, emotion and self came to neglect topics, this time neurophysiology and habituation, which James had shown to be intimately related to them. In addition, with ever-increasing specialisation within psychology, researchers came to ignore the relations among consciousness, thinking, emotion and self, which James had highlighted. As a result, it is only in the past few decades, with the advent of ‘interdisciplinary studies’, that cognition and emotion have been studied once again in terms of their significant overlap, as they were by James more than 100 years ago. And after a similarly long lapse, the role of attention is again a vital topic of research, even leading to explorations of its relation to will, which was a central interest of James.

In this context, returning to James's work can yield dividends, for there are many other insights that James has to offer; this is also true with regard to James's philosophical work, but that is beyond the scope of this essay. Because those insights often entail connections between different subfields of psychology, my own recent book on James's *Principles of Psychology* focused intentionally on some of the more important relations within his thought: between his treatments of mind and body, habit and thought, perception and conception, imagination and memory, cognition and emotion, consciousness and subconsciousness, attention and will, and self and others, not to mention psychology and philosophy, all of which make his work so timely to explore.

The revisions James made in traditional empirical psychology involved changing the Lockean model of the mind from the passive mode, as exemplified in Herbert Spencer's work, to the active mode, as accomplished by the introduction of subjective interest and individual choice into psychological dynamics, a crucial addition that James traced to insights from the British philosopher Shadworth Hodgson as well as to his own American mentor Chauncey Wright. James's subsequent radical empiricism, which included subjective experience as its leavening element, placed James at odds with the kind of mechanistic psychology that was later advanced by John B. Watson and all those who banished subjectivity from psychology.

With this in mind, we can consider one possible contribution that James's non-mechanistic psychology could make to current understanding of the relations between psychiatric conditions – in particular, depression, anxiety and panic of the sort suffered by James himself in a critical period of his life – and consciousness. To do so, we need to be aware of some additional facts, the relevant episode in James's life and the role played by two additional British people, the 17th century writer John Bunyan and the mid-19th century physiologist William B. Carpenter. Bunyan, as we shall see, suggested to James a practice that changed his life for the better – in fact, a practice that saved his life, as far as he was concerned – and Carpenter proposed an idea that helped James explain the efficacy of that practice, thus influencing his construction of a psychology that began with a focus on brain processes but ended with an affirmation of consciousness, indeterminacy and free will: not a radically free will independent of natural conditions, but a will possessing sufficient personal agency to make life worth living, for James and many others. James's notion of will, of voluntarism, of being able to make a difference in the world by virtue of his own capacities for resistance, participation and collaborative creation, was grounded upon his conviction that we live in a moral universe in which human action, however limited and pressed upon, can still have some sway, with each of us potentially ‘in the game’, as he put it. For if human life is truly the struggle that it feels like, as James remarked, it is vitally important for us to believe that the results of that struggle are not foreordained. The difference each of us makes may be small, but it nonetheless makes all the difference for us.

James struggled in his 20s and 30s with poor health, depression, neurasthenia, suicidal thoughts and other related conditions, both before and during the first years of his commitment to the development of psychology. His reading of the French philosopher Charles Renouvier, along with his reading of Wordsworth and Browning, helped him survive and intermittently to emerge from his depressive state. (Less well known, but also significant, is the role that his meditations on Shakespeare's *Hamlet* played in the alleviation of his condition.) Not surprisingly, the variety of causes that fed his depression and ill health are somewhat less understood. He himself feared that inherited physiological factors were at the root of his problems. This generated an understandably fatalistic anxiety, exacerbated by his inclination to accept the deterministic assumptions that undergirded modern science. This nexus of anxiety and supposition led him to the unhappy conclusion that ‘we are nature through and through’ and that ‘not even a wiggle of our will’ occurs without some cause outside of our control. This threw into jeopardy his earlier hope to ‘leave a trace’ in human history, one that only he (through his own self-determination) could leave. And it made him question the very possibility of what he called ‘the moral business’ by which he had hoped to make his ‘nick’, thereby contributing his small but real ‘mite’ to the common good. As he agonised about all of this, he realised that what was at stake was the thought of ‘my having a will’. Renouvier had offered an argument, effective for James only on an off-and-on-again basis, that no one can prove or disprove free will, but if free will does exist, its verification would lie in the personal act of freely willing to believe in it. Knowing that he was miserable when he did not believe in it, James committed himself to believe for a year and observe whether that belief made a positive difference in his life.

This is all background to an experience of massive anxiety and panic that was a turning point in James's life, almost certainly in 1870. This momentous incident was described, as if drawn from someone else's experience, in his *Varieties of Religious Experience*. It occurred, according to this ‘anonymous report’, during a period of ‘the worst kind of melancholy’ that took ‘the form of panic fear’. It consisted of ‘a horrible fear of my own existence’ that ‘came out of the darkness’, accompanied by ‘the image of an epileptic patient whom I had seen in the asylum, a black-haired youth with greenish skin, entirely idiotic, who used to sit all day on one of the benches…with his knees drawn up against his chin’, moving ‘nothing but his black eyes and looking absolutely non-human’. And here was the barb at the centre of his panic: ‘This image and my fear entered into a species of combination with each other. *That shape am I*, I felt, potentially. Nothing that I possess can defend me against that fate, if the hour for it should strike for me as it struck for him’. The horror of him and the fear for himself – and the sense of his own ‘merely momentary discrepancy from him’ – left James ‘a mass of quivering fear…with a horrible dread at the pit of my stomach’ that he managed to escape only by clinging to scripture texts like ‘The eternal God is my refuge’, ‘Come unto me, all ye that labor and are heavy-laden’, ‘I am the resurrection and the life’, and so forth. At the end of his description of this harrowing incident, James asserted: ‘Without clinging to these phrases rather than submit to the image of the idiotic patient, I think I should have grown really insane’.

I have written two articles about this episode, one providing new information about its immediately precipitating cause (namely, James's reading of the philosophy of Arthur Schopenhauer, with its pessimistic message about the illusion of individual personhood and of the associated belief in the indeterminacy of individual will) and the other identifying the source of its resolution. How James came to this resolution and how he came to understand and integrate its significance into his psychology is an interesting and relevant story.

In the months preceding the probable date of his fearful encounter with the idiotic, green-skinned phantom, James was not only depressed, but also reading John Bunyan's *Pilgrim's Progress* (1678–1684).^5^ As discovered fairly recently, his mother had given him a copy of this book (in a revised and simplified edition) in late January 1870. She did so purposefully, one assumes, since James was then in the midst of the same bout of suffering that eventually led to his ‘touching bottom’ during his hallucinatory panic attack. But in any case, James had clearly read the chapter entitled ‘The Fight’ in this revised version of Bunyan's work, as evinced by a dog-eared page within an extended passage that begins with Christian being ‘full of fear’ as he is confronted by the ‘foul fiend’ Apollyon, and then suffers even greater fear as he approaches the Valley of the Shadow of Death. At this point, Christian has a dream, not unlike James's own apparition, in which he comes to the edge of the ‘dark as pitch’ Shadow of Death. There he sees ‘ghosts and imps and fiends of the pit’, and hears ‘howls and yells as of men in great pain, who sat bound in woe and chains’. Despite his terror, Christian trudges on, seeing and hearing ‘dread things’ until ‘at last’ he hears ‘a band of fiends’ coming to torment him. In trembling fear, he considers ‘what he had best do’. And here, on that dog-eared page in James's recently discovered personal copy, is where Christian's tale touches James's life:

At times he had half a thought he would go back; but then he knew that he might be half way through the vale. He thought, too, of all that he had gone through, and that it might be worse to go back than to go on. So he made up his mind to go on, but the fiends drew near. But when they had come at him, as it were, he cried out with all his might, ‘I will walk in the strength of the Lord God’. (pp. 94–95)

Then, as Christian went on with his mind riveted on the strength of God, he was comforted by a voice saying, ‘Though I walk through the Valley-of-the-Shadow-of-Death, I will fear no ill, for thou art with me’. And with that, shrouded by these protective verses from Psalms 71 and 23, Christian ‘came to the end of the vale’.

It is not difficult to connect Christian's experience with James's. Similarly full of fear, James confronted his own nightmarish apparition and escaped his own spectre of damnation through the recitation of biblical verses, just like Christian. Although his fear of impending insanity was different from Christian's fear of punishment by ‘fiends of the pit’, the analogy is easy to see. And James himself, always a perceptive and thoughtful reader, would have seen it. Indeed, in a letter to his brother Henry on 7 May 1870, he wrote that ‘I have I think at last begun to rise out of the slough of the past 3 months’. Slough is, of course, a clear reference to the ‘Slough of Despond’ in *The Pilgrim*'*s Progress*.

We can now consider how James translated this experience into a usable psychological proposition: how, in psychological terms, repeating biblical phrases helped James endure his journey through the Valley of the Shadow of Death without utterly breaking down. This is where the work of William B. Carpenter enters the story. Four years after his devastating experience, as he was beginning to formulate his own psychology, James read Carpenter's *Principles of Mental Physiology* (1874).^6^ In a published review, he specifically noted Carpenter's notion of ‘ideo-motor action’, which applied to some ‘curiosities of our mental life’, instances in which a dominating (we would say obsessive) idea gives rise to repetitive motor (behavioural) actions. What James came to realise was that ideo-motor action is, in fact, a more general principle of mental life, not confined to idiosyncratic clinical cases. Indeed, he concluded that the evolutionary function of all ideas is precisely to serve as intermediaries between sensory stimulation and behavioural movement, all of which occur without the intervention of the will. Ideas are naturally ‘impulsive’, as James put it. So the question is not why ideas lead to action (sensation-ideation-action being the normal course of events), but rather, why some ideas do not.

This is where James's Bunyan-related experience melded with his Carpenter-inspired realisation. Why had the image of the idiotic patient not led to a breakdown in his sanity as James had fully expected? He came to believe that the impulsive efficacy of the image was thwarted when his mind was distracted by more powerful ideas: when images associated with the biblical phrases became more dominant in his consciousness by means of his wilfully attending (or ‘clinging’) to them. James was well aware that the mind sometimes entertains multiple ideas, with the strongest taking precedence over the weaker ones. Now he added that selective attention could change the relative strength of an idea, bringing it to greater intensity in the centre of consciousness, while other ideas recede as a consequence to the margins of awareness. In short, James made Carpenter's observation about occasional ideo-motor action into a full-blown ideo-motor theory, with an important codicil about how ideas come to be, or not to be, in the centre of one's consciousness. This is precisely where subjective interest and wilful attention come into play, James concluded. Will, in this rendition, is equivalent to enhanced selective attention, which is directed by our interests. (James later clarified that our interests can be aesthetic and moral as well as intellectual and practical.) Will creates nothing; it does not directly affect action, it can only do so indirectly by increasing the prominence of one idea over others, thereby ‘loading the dice’ for one action over others. (Another way to say this is that we cannot will a movement independently of thinking about it.) In this way only do dominant ideas bring about ‘voluntary action’ as opposed to involuntary, instinctive or habituated responses.

This psychological explanation, giving a restricted yet significant role to personal interest and selective attention, provides the essential framework for James's chapter on ‘The Will’ in his *Principles of Psychology*. In that chapter, he explains and defends his ideo-motor theory and outlines ‘Five Types of Decision’ according to the prominence, conflict or absence of competing ideas. He also discusses extreme cases of ‘The Explosive Will,’ in which the impulsive power of ideas is not sufficiently repressed by countervailing ideas, and ‘The Obstructed Will,’ in which the repression of ideas is excessive. Clearly, what James offered was primarily a phenomenological description of the experience of will. Simple though it is, it bears consideration as contemporary research re-opens the matter of selective attention and its relation to will or willpower. The proof of its theoretical adequacy will depend, of course, upon the accumulation of scientific evidence, but its practical utility will be judged best by psychotherapeutic outcomes. Can a focus on certain ideas or images facilitate behavioural change, whether immediately or after repeated occurrence, whatever might be going on ‘behind the scenes’ in terms of biochemical transformations and neurological processes? Research on meditation suggests that it can. What about therapeutic success? More should be made of this, especially among those who typically emphasise biomedical factors in treatment settings. More particularly, it might be useful to explore James's claim that, although individuals cannot directly will a change in their psychiatric condition, they can and should maintain a focus on the idea of an alternative, keeping that idea forcefully in mind during the course of whatever kind of therapy might be taking place. It cannot hurt, and it might well prove to be beneficial. A good deal of research, after all, has shown that the mind is much more powerful, in a variety of ways, than was once assumed.

This is all reminiscent of Viktor Frankl's comment, very possibly made with James's views in mind, that even when all other means of changing a situation are blocked, as in a concentration camp or a severe psychiatric condition, one can at least posit one's own attitude toward what is happening in one's life.^7^ That attitude, expressing a firmly held idea or wish for another state of affairs, may well contribute, along with other remedies, to a positive change. William James, the grateful recipient of many British ideas, certainly thought so.

Interested persons can find additional information about James and his work, relevant to this essay, in references^8^^,^^9^^,^^10^^,^^11^. The title of the last reference, ‘A moralist in an age of scientific analysis and skepticism', is used to describe James, but it comes, in fact, from James’s own description of the British novelist George Eliot, indicating yet another link between James and British authors.

## About the author

**David E. Leary** is University Professor Emeritus at the School of Arts and Sciences, University of Richmond, USA.
